# Mouse Rif1 is a regulatory subunit of protein phosphatase 1 (PP1)

**DOI:** 10.1038/s41598-017-01910-1

**Published:** 2017-05-18

**Authors:** Rasa Sukackaite, Daniela Cornacchia, Malene Ringkjøbing Jensen, Philippe J. Mas, Martin Blackledge, Elin Enervald, Guangyou Duan, Tania Auchynnikava, Maja Köhn, Darren J. Hart, Sara B. C. Buonomo

**Affiliations:** 1 European Molecular Biology Laboratory, Grenoble Outstation, Grenoble, 6 rue Jules Horowitz, 38042 France; 2 European Molecular Biology Laboratory, Monterotondo Outstation, Adriano Buzzati-Traverso Campus, Via Ramarini 32, 00015 Monterotondo, Italy; 3 Institut de Biologie Structurale (IBS), CEA, CNRS, Université Grenoble Alpes, 38044 Grenoble, France; 4 Genome Biology Unit, European Molecular Biology Laboratory, Meyerhofstrasse 1, 69117 Heidelberg, Germany; 5 Wellcome Trust Center for Cell Biology, University of Edinburgh, Michael Swann Building, Max Born Crescent, Edinburgh, EH9 3BF UK; 6 Thermo Fisher Scientific Baltics, UAB, Graiciuno 8, 02241 Vilnius, Lithuania; 7 Memorial Sloan Kettering Cancer Center, Center for Stem Cell Biology, Lorenz Studer Group, Rockefeller Research Laboratories, 430 E 67th Street, 10065 New York, NY, USA; 8 Institut de Biologie Structurale (IBS), CEA, CNRS, Université Grenoble Alpes, 38044 Grenoble, France; 9 Institute of Cell Biology, School of Biological Sciences University of Edinburgh, Roger Land, Building, Alexander Crum Brown Road, Edinburgh, EH9 3FF UK; 10 School of Life Sciences, Qilu Normal University, Wenbo Road 2, 250200 Jinan, China; 11 Faculty of Biology and Centre for Biological Signalling Studies (BIOSS), University of Freiburg, Schänzlestr 18, 79104 Freiburg, Germany

## Abstract

Rif1 is a conserved protein that plays essential roles in orchestrating DNA replication timing, controlling nuclear architecture, telomere length and DNA repair. However, the relationship between these different roles, as well as the molecular basis of Rif1 function is still unclear. The association of Rif1 with insoluble nuclear lamina has thus far hampered exhaustive characterization of the associated protein complexes. We devised a protocol that overcomes this problem, and were thus able to discover a number of novel Rif1 interactors, involved in chromatin metabolism and phosphorylation. Among them, we focus here on PP1. Data from different systems have suggested that Rif1-PP1 interaction is conserved and has important biological roles. Using mutagenesis, NMR, isothermal calorimetry and surface plasmon resonance we demonstrate that Rif1 is a high-affinity PP1 adaptor, able to out-compete the well-established PP1-inhibitor I2 *in vitro*. Our conclusions have important implications for understanding Rif1 diverse roles and the relationship between the biological processes controlled by Rif1.

## Introduction

Rif1 protein is conserved throughout evolution from yeast to mammals. It was initially identified in *Saccharomyces cerevisiae* as regulator of telomere length^1–4^, a function that however appears to be specific to yeast. In fact, no major telomeric role could be attributed to mammalian Rif1^5^, which instead has been implicated in various aspects of DNA repair^5–12^. Our group and others have recently identified Rif1 as a key regulator of DNA replication timing, which is the first common function of this protein across species^13–17^. In addition, we showed recently that mouse Rif1 is involved in the organization of the three-dimensional (3D) contacts of replication timing domains at around the same time when the replication-timing program is established in G1^18^. These data raise the question of whether Rif1-dependent control of replication timing is related to its architectural function. In fact, the molecular mechanism through which Rif1 mediates such diverse processes remains unclear.

The analysis of Rif1 protein structure provides only limited clues to its mode of action. Mammalian Rif1 contains two conserved regions that could potentially mediate protein-protein and protein-nucleic acid interactions: an N-terminal region that is predicted to comprise HEAT-type α-helical repeats (HEAT repeats) and a disordered tripartite C-terminal region, comprising CRI, CRII and CRIII subdomains (Supp. Fig. 1A). With the exception of CRIII, all these domains are conserved^11, 19, 20^. The HEAT repeats are required for mouse Rif1 recruitment to double strand breaks^21^ and the mammalian CRII is a functional DNA-binding domain that selectively binds to cruciform DNA^20, 22^. Fission yeast Rif1 was recently reported to bind G quadruplexes^23^, however, the DNA-binding domain is still to be localized. The budding yeast homolog of the mammalian Rif1 CRII-domain mediates the interaction with Rap1 and DDK^15^, and is also required for Rif1 tetramerization^4^. Mammalian Rif1 also multimerizes through its C-terminal domain, although a finer mapping of interacting regions has not been performed^20^.

Throughout evolution, Rif1 has maintained one or more motifs that potentially mediate the interaction with the Ser/Thr protein phosphatase 1 (PP1), such as the PP1 docking motif [K/R][V/I]xF and the conserved SILK sequence, an additional binding motif necessary for a subset of PP1-interacting proteins^19, 24, 25^. In budding yeast, Rif1 PP1 docking motifs are localized at the N-terminus of the protein. Their function has been validated through mutagenesis, demonstrating that Rif1 counteracts DDK activity on the replicative MCM4 helicase through its interaction with PP1^15, 26, 27^. Also, human and Drosophila Rif1 homologs have been identified in PP1-associated complexes^28–30^. However, human and mouse Rif1s contain at least two different potential PP1-interacting peptides, canonical RVSF/SILK motifs in CRI and an additional KIAF motif in the HEAT repeats. These structural features indicate that the interaction of Rif1 with PP1 may represent a central aspect of Rif1 biological role throughout evolution. However, which of these mammalian Rif1 motifs mediates the binding to PP1, how and which biological function of Rif1 requires this association, remains to be determined.

To understand the molecular basis of Rif1 multiple functions, we sought to define the composition of cellular Rif1 complexes. Due to the lack of suitable antibodies and biochemical difficulties in isolating Rif1, no comprehensive study of the endogenous Rif1-associated complex has so far been published. The data available are in fact limited to soluble complexes^20, 31^. Given the association of at least half of Rif1 to insoluble nuclear structures^13, 17^, investigating its function is challenging. In proof-of-principle studies, DamID-based approaches have recently identified Rif1 in close proximity to LaminA/C^32^. Here, we characterize the protein complexes associated with endogenous Rif1 in mouse cells, including those found in the insoluble fraction. Among its interactors, we focused on protein phosphatase 1 (PP1), given the compelling evidence for a functional significance of this interaction. We have mapped the interaction surface in Rif1 CRI and have generated point mutations that effectively abolish it. The interaction is nanomolar affinity but does not mask the PP1 active site. Our data therefore classify Rif1 as a *bona fide* PP1 regulatory subunit.

## Results

### Rif1 associated protein complexes

The characterization of endogenous Rif1-associated complexes has been hampered by the lack of optimized antibodies and the subnuclear localization of this protein, which render it extremely challenging to isolate. In this respect, we showed that Rif1 is associated to the nuclear lamina^13, 18^ and binds the genome at regions of constitutive heterochromatin^18^. Such features notoriously confer high protein insolubility. Accordingly, conventional approaches for the isolation of Rif1 would require harsh conditions such as high detergent or salt concentrations that result in loss of molecular interactors. To circumvent these limitations, we have optimized a biochemical extraction protocol employing low detergent and physiological salt concentrations, thereby preserving interactions whilst achieving solubilisation of the nuclear lamina-associated fraction (Supp. Fig. 1B,C,D and E). In addition, the use of cells derived from our previously described Rif1^FLAG-HA2^ (Rif1^FH^) mouse line^13^ permitted Rif1 affinity purification using standardized antibodies for immunoprecipitation.

**Figure 1:**
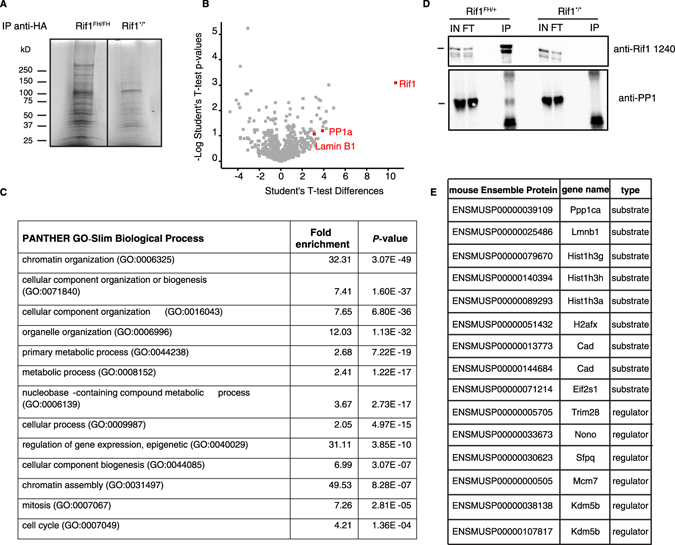
Rif1-associated complexes are enriched for chromatin-related proteins and PP1 substrates/regulators. (**A**) Immunoprecipitation of Rif1-associated proteins from extracts obtained from ESCs homozygous for Rif1^FH^ or Rif1^+^ alleles, visualized by Coomassie blue-stained SDS-PAGE. (**B**) Determination of Rif1 interactome using label-free quantitation pipeline MaxQuant^49^. For volcano plot, *t*-test was performed on data from 3 Rif1^FH/+^ and 2 Rif1^+/+^ ESC independent lines analysed in 2 independent experiments. *t*-test difference ratios were plotted against the negative logarithmic *P*-value of the *t*-test. Rif1, Lamin B1 and PP1 LFQs are indicated in red. (**C**) Top gene ontology (GO) terms enriched within nuclear proteins associated to Rif1^FH^, listed in supplemental table C (cut-off of enrichment over negative control 1.5). The analysis is a Panther overrepresentation test against the complete GO biological process annotation dataset. Bonferroni correction was applied. (**D**) PP1 interaction with Rif1 was confirmed by immunoprecipitation of Rif1-associated proteins from Rif1^FH/+^ ESCs and immunoblotting with anti-PP1 antibody. IN = input; FT = flow through; IP = immunoprecipitated. (**E**) List of the known PP1 substrates/regulators identified in the Rif1-associated complexes. The Rif1 interactome enrichment for PP1 substrates/regulators is statistically significant as evaluated by Fisher’s exact test (***P***-**value**: 0.00111).

To gain further molecular insight into Rif1 function we employed a proteomic mass spectrometry approach to determine the composition of the Rif1-associated complexes from mouse embryonic stem cells (ESCs) expressing a heterozygote Rif1^FH^ (Fig. 1A,B and Supp. Table 1A, total proteins). This approach has identified 96 nuclear interactors associated with Rif1 (Supp. Table 1C, above 1.5 nuclear). Consistent with the multiple aspects of nuclear organization and chromatin metabolism in which Rif1 has been implicated, the identified interactors were significantly enriched in chromatin organization and assembly and nuclear (organelle) organization (Fig. 1C). Within the top 15 Rif1 partners (Table 1) we found lamin B1, confirming our recent report^18^, and PP1 (highlighted in Fig. 1B and D). Interestingly, the proteins identified in the Rif1-associated complexes are also significantly enriched in known PP1 substrates and regulators (Fig. 1E). This observation, together with genetic evidence from yeast^15, 26, 27^ and Drosophila^16^, suggests that Rif1 may itself be a regulatory subunit of PP1. To explore this hypothesis, we have further characterized the Rif1-PP1 interaction in molecular detail.

**Table 1:** Top Rif1 nuclear interactors, based on the levels of LFQ enrichment over negative control.

Protein ID	Extended name	Function
Q6PR54|RIF1	Rap1-associated protein.	DNA replication, DNA repair.
P14206|RSSA	40S ribosomal protein SA.	Required for the assembly and/or stability of the 40S ribosomal subunit.
P62137|PP1A	Protein phosphatase 1 subunit A.	Ser/Thr phosphatase with essential roles in cell proliferation, DNA repair and development.
P23116|EIF3A	Eukaryotic translation initiation factor subunit A.	RNA-binding component of the eukaryotic translation initiation factor 3 (eIF-3) complex.
P60229|EIF3E	Eukaryotic translation initiation factor subunit E.	Component of the eukaryotic translation initiation factor 3 (eIF-3) complex.
P14733|LMNB1	Lamin B1.	Structural component of the nucleus, part of the nuclear lamina.
P21619|LMNB2	Lamin B2.	Structural component of the nucleus, part of
O88685|PRS6A	26S proteasome regulatory subunit 6A.	Regulatory subunit of the 26 proteasome.
P43346|DCK	Deoxycytidine Kinase.	Required for the phosphorylation of the deoxyribonucleosides deoxycytidine (dC), deoxyguanosine (dG) and deoxyadenosine (dA).
P62806|H4	Histone H4.	Core nucleosome component.
Q9WVJ2|PSD13	Proteasome (Prosome, macropain) 26S subunit, non-ATPase, 13.	Regulatory subunit of the 26 proteasome.
P46935|NEDD4	E3 ubiquitin-protein ligase NEDD4.	Accepts ubiquitin from an E2 ubiquitin-conjugating enzyme and then directly transfers the ubiquitin to targeted substrates.
Q6P5F9|XPO1	Exportin 1.	Mediates the nuclear export of cellular proteins (cargos) bearing a leucine-rich nuclear export signal (NES) and of RNAs.
P68040|RACK1	Receptor for activated protein kinase C.	Involved in the recruitment, assembly and/or regulation of a variety of signaling molecules.
P34022|RANG	Ran-specific GTPase-activating protein.	Inhibits GTP exchange on Ran.

### Rif1 interacts with PP1 through canonical C-terminal RVSF and SILK motifs

PP1 has no intrinsic specificity for its substrates, but interacts via regulatory subunits that contain defined docking motifs. In addition, they are often intrinsically disordered proteins (IDPs) whose folding follows the interaction with PP1 itself. Several regulatory subunits are known to target PP1 to chromatin, including NIPP^33^, PNUTS^34^, RepoMan^30^ and Ki67^35^. Remarkably, recent mapping of PP1 genome-wide occupancy has shown very limited overlap between PP1 binding sites and the sites occupied by the known regulatory subunits, indicating that many more remain to be discovered^36^.

**Figure 2:**
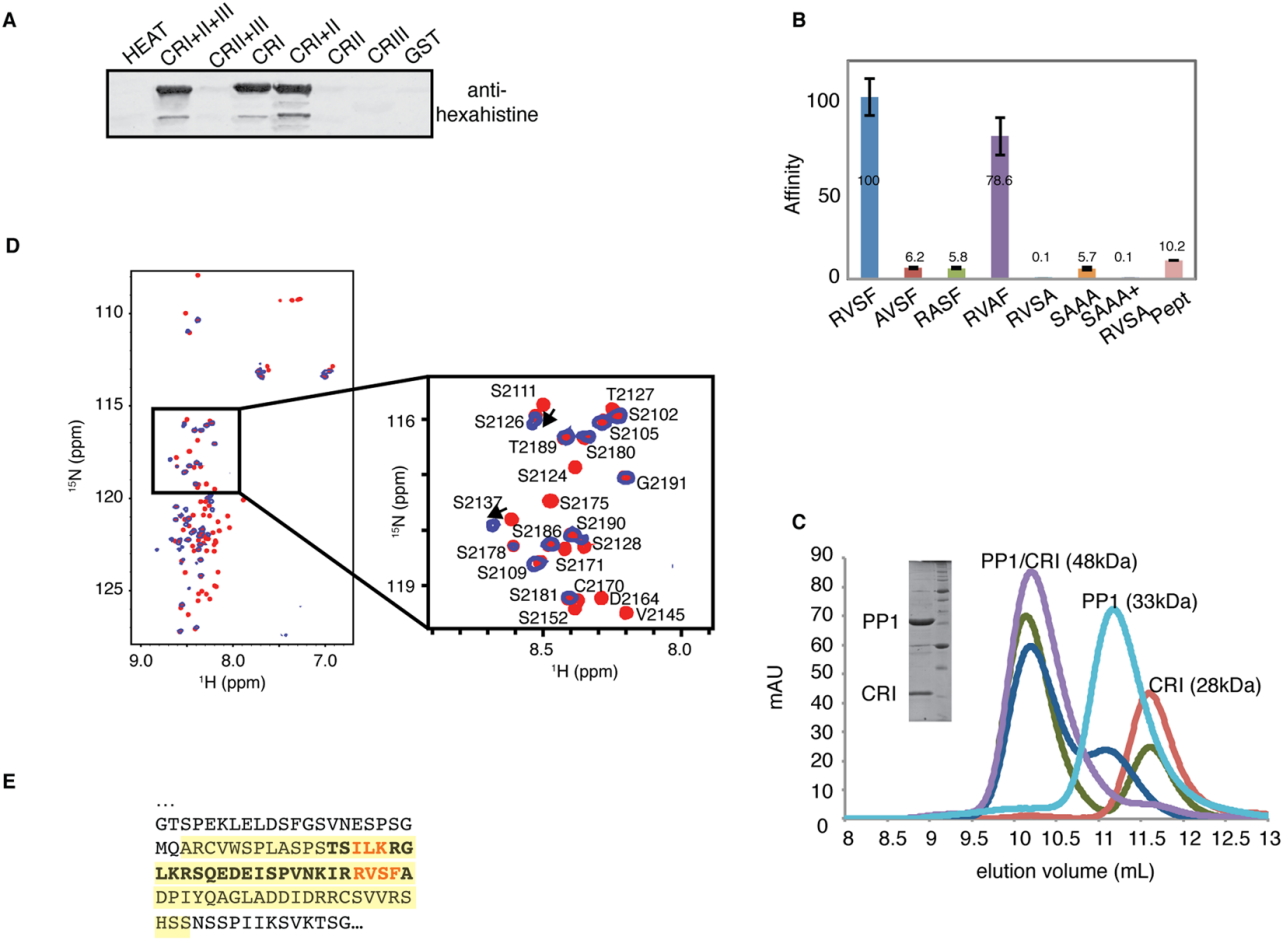
The Rif1 CRI region contains two canonical RVSF and SILK motifs interacting with PP1. (**A**) Beads loaded with GST-Rif1 fragments were incubated with cell lysates containing hexahistidine-PP1. PP1 retained by means of interaction with Rif1 was eluted and analysed by imunoblotting using antibodies against the hexahistidine tag. (**B**) The affinity of Rif1 CRI mutants and peptide for PP1 was determined by the SPR and expressed as percentage of wild-type. SAAA indicates the mutation of the residues within the SILK motif. (**C**) Analysis of Rif1 CRI interaction with PP1 by size exclusion chromatography. PP1 (light blue), Rif1 CRI (red), PP1/CRI at molar ratio 1:1 (magenta), PP1/CRI at molar ratio 1:3 (green) and PP1/CRI at molar ratio 2:1 (blue) were subjected to analytical gel-filtration. The Coomassie-stained gel shows recombinant PP1 and Rif1 CRI co-eluting from the column. mAU = milli Absorbance Unit. (**D**) Superposition of the ^1^H-^15^N HSQC spectra of ^15^N-labeled CRI (red) and ^15^N-labeled CRI in the presence of PP1 (blue). In the inset, assignments are shown for CRI for a selected region of the HSQC spectrum. (**E**) PP1-interacting region in mouse Rif1. Interacting residues identified by NMR analysis are highlighted in yellow, residues present in the synthetic peptide (Pept) are shown in bold, and residues subjected to mutagenesis are shown in red.

Sequence analysis suggests potential PP1 docking motifs in both CRI at the mouse Rif1 C-terminus (SILK and RVSF, residues 2128–2131 and 2150–2153) and within the N-terminal HEAT repeats (KIAF, residues 291–294) (Supp. Fig. 1A). To identify which of the motifs mediate a functional interaction, GST-Rif1 fragment fusion proteins were bound to glutathione beads (Supp. Fig. 1F and G) and incubated with an *E. coli* crude lysate containing recombinant hexahistidine-tagged PP1. PP1 co-precipitated with Rif1 CRI+II+III, CRI+II and CRI and did not bind fragments lacking CRI (CRII, CRIII, CRII+CRIII and HEAT repeats) (Fig. 2A). These results show that Rif1 CRI is a PP1-binding region. To measure the contribution of individual CRI residues to PP1 binding, we substituted every residue in the primary motif RVSF, and ILK residues in the additional binding motif SILK for alanine. Purified wild-type and mutant Rif1 CRI proteins were injected over immobilized recombinant purified α-isoform of mammalian PP1 in a surface plasmon resonance (SPR) assay. Analysis of binding curves revealed that the RVSF to RVSA substitution had the most pronounced effect on PP1 binding, resulting in less than 0.1% of the affinity of the wild-type interaction (Fig. 2B). The RVSF to RASF and SILK to SAAA substitutions resulted in approximately 20-fold decrease in affinity, suggesting a role of these residues in PP1 binding. Alanine substitution of the non-conserved serine residue in the canonical motif (RVSF to RVAF) had only a minor effect on PP1 binding. In contrast to the result on the longer CR1 construct (residues 2093–2190), a minimal synthetic peptide comprising only residues 2127–2154 of mouse Rif1 showed only 10% of binding affinity, indicating that residues in the natural protein beyond the core motifs may be involved in the interaction (Fig. 2B, Pept.). In conclusion, the single RVSF to RVSA substitution strongly inhibits PP1 binding. This can be further combined with the SAAA variant of the SILK motif to produce a Rif1 variant that does not detectably bind PP1 (Fig. 2B, SAAA+RVSA).

Previously, using the random library approach ESPRIT^37^ we identified a high-yielding soluble fragment encompassing mouse Rif1 CRI (residues 2093–2190)^22^. This protein exhibits behaviours characteristic of IDPs: it migrates at a higher-than-expected molecular mass on SDS-PAGE (15 kDa instead of 10.8 kDa, Fig. 2C), exhibits a higher Stokes radius by gel-filtration than standard globular proteins of similar molecular mass (elutes as 28 kDa, Fig. 2C), and is resistant to heat-induced aggregation^22^. The two-dimensional ^1^H-^15^N HSQC NMR spectrum of CRI shows very little chemical shift dispersion in the ^1^H^N^ dimension, confirming that it is indeed intrinsically disordered (Fig. 2D and ref. 22). We used this fragment to perform interaction studies with PP1. Recombinantly expressed and purified PP1 (37.5 kDa) elutes from a gel-filtration column as a single peak with apparent molecular weight of ~33 kDa (Fig. 2C). In the presence of Rif1 CRI at the molar ratio 1:1, both the proteins were eluted in a single peak with apparent molecular weight of ~48 kDa. The excess of PP1 or CRI resulted in two peaks: the ~48 kDa peak corresponding to the protein complex and the other peak corresponding to the excess protein. These results indicate that Rif1 CRI binds to PP1 in a 1:1 ratio. Next, we compared spectra of ^15^N-labeled CRI recorded in the presence and absence of unlabelled PP1. A significant fraction of the resonances disappeared from the spectrum in the presence of PP1, while others retained their chemical shift values or shifted slightly (Fig. 2D and Supp. Fig. 1H). The former correspond to the residues interacting directly with PP1; following assignment of the CRI spectrum they were identified as residues 2115–2178, encompassing the SILK and RVSF motifs (Fig. 2E). Interestingly, the NMR signals of R2136, S2137, and Q2138 in the centre of this region were visible, although with reduced intensity, indicating that these residues stay sufficiently flexible in the CR1-PP1 complex to give rise to solution NMR signals. PP1 regulatory subunits are typically IDPs, further strengthening the conclusion that Rif1 is a PP1-regulatory subunit.

### Rif1 CRI binds to PP1 with high affinity

**Figure 3:**
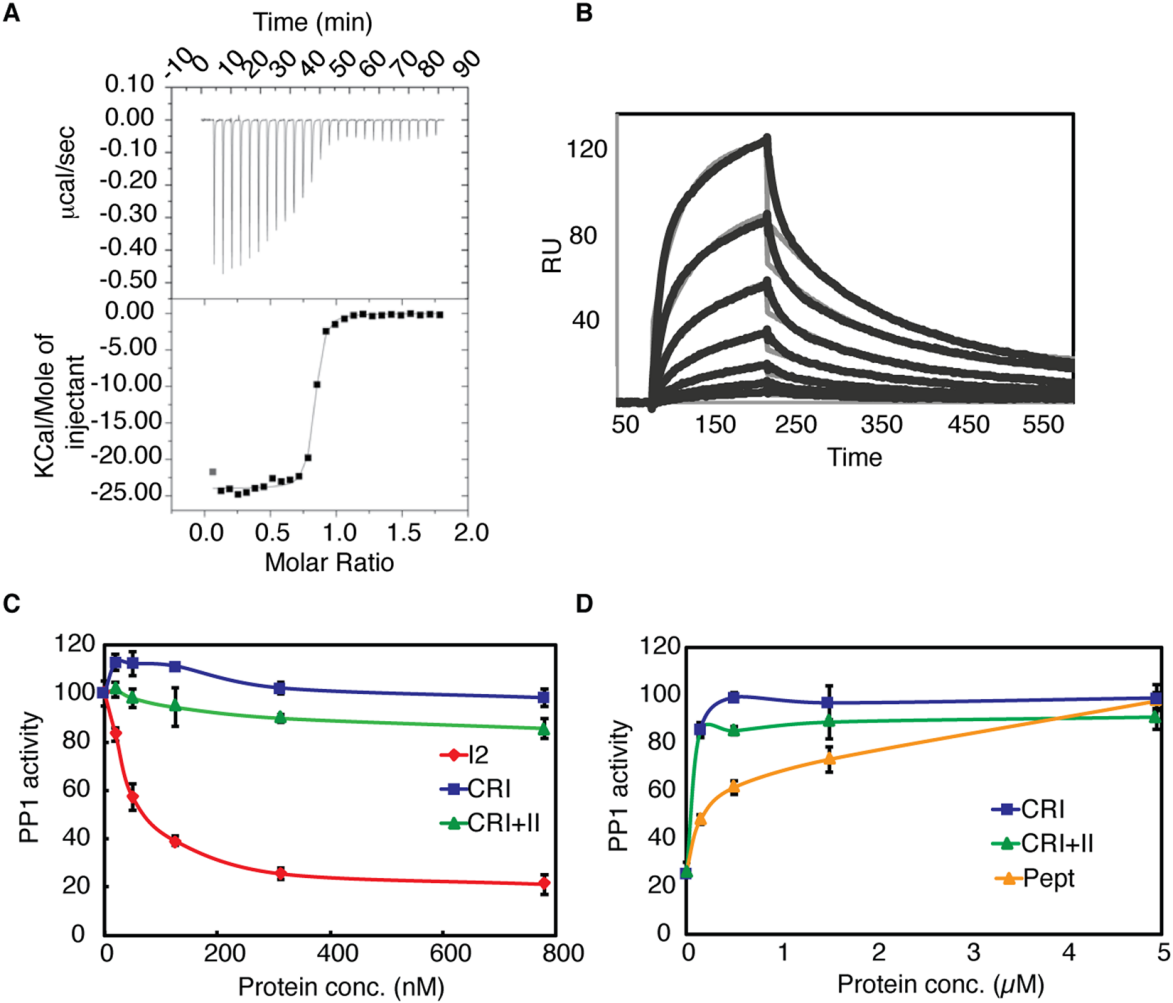
Rif1 CRI mediates high-affinity binding to PP1. (**A**) ITC of PP1 and Rif1 CRI was used to quantify the affinity of the interaction (K_D_ = 22.8 ± 4.2 nM). (**B**) Kinetic analysis of Rif1 CRI-PP1 binding by SPR. CRI at 0.625, 1.25, 2.5, 5, 10, 20, 40 nM was injected in duplicate over captured PP1. The raw data (thick black lines) are overlaid with a global fit to a 1:1 binding model. (K_D_ = 9.7 ± 1.7 nM). (**C**) Dephosphorylation assay in presence of I2, Rif1 CRI and CRI+II. (**D**) Activation of I2 inhibited PP1 (PP1/I2 at molar ratio 1:3.75) by Rif1 fragments and Pept. PP1 activity was calculated from initial reaction rates (Vo) determined measuring optical absorbance of dephosphorylated pNP product.

The concentration of PP1 regulators in the cell is higher than that of PP1 itself, suggesting that formation of different complexes and their balance is driven by competition for a limited phosphatase pool^38, 39^. Therefore, different affinities of interactors for PP1 likely regulate the balance between different complexes. To measure the strength of the Rif1 CRI-PP1 interaction, we employed isothermal titration calorimetry (ITC) and SPR. ITC of PP1 and Rif1 CRI confirmed the 1:1 binding with K_D_ = 22.8 ± 4.2 nM (∆H = - 23.8 ± 0.129 kcal/mol; ∆S = −42.4 ± 4.1 cal/mol/deg, Fig. 3A). Injections of various concentrations of CRI over PP1 immobilized on a CM5 sensor chip resulted in sensorgrams with a clear association phase followed by a slow dissociation phase (Fig. 3B). Binding curves were fitted using the simple Langmuir binding model 1:1 with K_D_ = 9.7 ± 1.7 nM. The nanomolar affinity of Rif1 CRI to PP1 is close to the highest values reported for PP1 interactors PNUTS (K_D_ = 8.7 nM)^40^ and spinophilin (K_D_ = 9.3 nM)^25^, while the determined K_D_ values for NIPP (73 nM)^41^ and GADD34 (62 nM)^42^ were significantly weaker.

Binding of regulatory proteins to the RVxF interaction site, which is approximately 20 Å from the PP1 catalytic site, does not alter the PP1 catalytic function; however, a number of PP1 regulators can inhibit phosphatase activity^43^. The best-characterized inhibitory regulator is Inhibitor 2 (I2), which has three PP1 docking sites: RVxF, SILK and IDoHA. The latter binds as an α-helix, and blocks phosphatase activity by covering the catalytic site^44^. To analyse the effect of interaction on PP1 activity we measured phosphatase activity of PP1 in the presence of various concentrations of Rif1 CRI and CRI+II using I2 for comparison. Rif1 fragments had no effect on pNPP substrate hydrolysis by PP1, while I2 showed potent inhibition at nanomolar concentrations (Fig. 3C). This indicates that Rif1-C does not block PP1 activity by covering the active site. Moreover, addition of recombinant Rif1 fragments to PP1 in presence of I2 fully restored PP1 activity (Fig. 3D). A synthetic peptide with lower affinity to PP1 than Rif1 CRI also activated PP1 (Fig. 3D); however, much higher concentrations of the peptide were needed, consistent with the lower affinity measured by SPR (Fig. 2B). These data suggest that Rif1-C, with its nanomolar affinity to PP1, may be able to compete effectively for binding to PP1 in the presence of other PP1 regulators.

## Discussion

Our data classify Rif1 as a *bona fide* regulatory subunit for PP1. The significant enrichment for chromatin-related proteins in the Rif1-associated complexes represents an important step towards the understanding of its multiple related functions. We have recently described the involvement of Rif1 in organizing nuclear architecture in G1^18^ and its function function as global regulator of replication timing. Experimental evidence had already connected these two processes, suggesting a role for nuclear 3D organization in the establishment of replication timing. Our results demonstrate that Rif1 links these two processes, yet the underlying molecular mechanism remains to be determined. Rif1 may act as a hub, controlling in parallel the two processes through de-phosphorylation of different substrates independently responsible for the control of nuclear architecture and replication timing. Alternatively, Rif1 could control replication timing at two levels, firstly by determining 3D chromatin contacts during G1 and influencing the timing of origin firing, possibly by regulating their affinity for limiting replication factors. Secondly, in S-phase, Rif1 may also mediate de-phosphorylation of proteins that control activation of origins of replication. In this case, the architectural function could be independent of the Rif1-PP1 interaction, potentially making the Rif1 mutants defective for PP1 binding described here a very useful separation-of-function allele.

Eukaryotic genomes encode a very limited number of phosphatases (about 40 Ser/Thr phosphatases in humans), intended to counteract the function of approximately 420 Ser/Thr kinases^45^. Consequently, each phosphatase is involved in a wide range of processes, where specificity is ensured by the interaction with regulatory subunits. Therefore, these represent the key to dissection of the individual roles of de-phosphorylation in different pathways. By inactivating a specific regulatory subunit, or inhibiting its interaction with phosphatase partners, it will be possible to interfere with a restricted subset of the phosphatase-mediated functions. In a first step towards this general approach, we believe that a comprehensive identification of PP1 substrates whose dephosphorylation depends upon Rif1, and characterization of these interactions at the molecular level, will elucidate the role of PP1 in the regulation of individual pathways. To further complicate the understanding of its functions, PP1 is present in three different isoforms, α, β/δ and γ1. Although more than 89% identical, these isoforms show distinct nuclear distribution^46, 47^, likely an important aspect of their functional specificity. Interestingly, previous work had identified that Rif1 preferentially associated to PP1α over PP1γ^30^. The data we present here confirm this finding, as we did not identify PP1γ as part of the Rif1-associated complex. Moreover, PP1α and Rif1 share a similar subnuclear distribution, being both associated to the insoluble nuclear structural components, unlike PP1γ^46, 47^. In the future, it will be interesting to understand the relationship between Rif1 and PP1 subnuclear distributions and how this contributes to substrate specificity.

## Methods

### ESCs chromatin isolation and Rif1 solubilisation test

Cell fractionation and solubilisation of chromatin-bound proteins by salt extraction was performed as described in ref. 48. For nuclease-mediated solubilisation of chromatin-bound proteins 2 × 10^6^ ESCs were used for each reaction. MNase digest: chromatin was incubated in MNase buffer with 1 U MNase (Sigma N3755)/100 μl reaction volume at 28 °C for 1,1.5 and 2 h. The reaction was stopped with 5 μl 200 mM EGTA. DNAse I digest: chromatin was incubated in DNAse buffer with 50 U DNAse-I (Roche, 04536282001)/100 μl reaction volume for 2 h at 37 °C. The reaction stopped with 5 μl 0.5 M EDTA. RNAse A digest: chromatin was incubated in RNase buffer with 2 μg RNase A (Sigma, R5250)/100 μl reaction volume for 2 h at 37 °C. The reaction was stopped by putting samples on ice. Double RNAse+DNAse digest: chromatin was incubated with 2 μg RNase A+50 U DNAse-I/100 μl reaction volume for 2 h at 37 °C. The reaction was stopped with 5 μl 0.5 M EDTA on ice. Benzonase digest: chromatin was incubated in 50 U Benzonase (Sigma, E1014)/100 μl reaction volume for 2 h at 37 °C. The reaction was stopped with 5 μl 0.5 M EDTA.

After nuclease digestion samples were divided in half for DNA or protein analysis. DNA samples were subjected to Proteinase K digestion (Proteinase K, Sigma P6556 1 h 37 °C) followed by DNA extraction by standard phenol-chloroform procedure and loaded onto a 0.7% agarose gel for electrophoresis. Laemmli sample buffer was added to the protein samples, to a final concentration of 1X, samples were boiled according to standard procedures and used for western blotting.

### Protein extract preparation

10^9^ Rif1^FH/+^ and Rif1^+/+^ mouse ESCs were harvested and resuspended in cold Hypotonic buffer (25 mM Tris HCl pH 7.4, 50 mM KCl, 2 mM MgCl_2_, 1 mM EDTA, freshly added Protease Inhibitors) at 20 000 cells/μl and incubated 20 min to eliminate the cytoplasmic fraction. A total of 3 Rif1^FH/+^ and 2 Rif1^+/+^ cell lines were used in 2 independent experiments. Samples were centrifuged 15 min at 1 500 g, 4 °C and supernatant was discarded. Nuclei were washed twice in cold Hypotonic buffer and resuspended in cold Benzonase buffer (50 mM Tris HCl pH 8.0, 100 mM NaCl, 1.5 mM MgCl_2_, 10% Glycerol freshly added Protease inhibitors) at 20 000 cells/μl. Samples were subjected to 3 cycles of snap freezing and thawing. Fifty U/ml of Benzonase (Sigma, E1014) was added to the thawed extracts and incubated at RT for 25 min. Triton X-100 was added to a final concentration of 0.2% and incubated on ice for 10 min. Samples were centrifuged for 20 min at 16 000 g to remove insoluble debris. Supernatants were used for immunoprecipitation (IP). One percent of the extract was used as Input and not subjected to IP.

### Immunoprecipitation (IP) of Rif1–associated complex

Rif1^FH^ and interacting factors were immunoprecipitated using Anti-Flag M2 affinity gel (Sigma, A2220) according to the manufacturer’s instructions. Protein extracts prepared as described above were pre-cleared with Sepharose beads (Sigma, 4B200) for 1 h at 4 °C. Two millilitres of anti-Flag resin was used for 50 ml extract and IP was performed overnight at 4 °C. Following incubation, beads were collected at 2 000 g for 5 min, 1% of the supernatant was retained as flowthrough and the rest was discarded. Beads were subjected to 3 washes for 5 min at 4 °C in cold IP buffer (50 mM Tris HCl pH 8.0, 150 mM NaCl, 1.5 mM MgCl_2_, 10% Glycerol, 0.1% Triton X-100 with freshly added Protease inhibitors). Rif1^FH^ was eluted by competition with Flag-peptide (Sigma, F4799) through 3 consecutive steps of incubation of the beads with 1 packed volume of Flag-peptide diluted in IP-buffer, for 1 h at 4 °C under rotation. Eluates were concentrated and excessive Flag-peptide was removed using centrifugal filter units (Amicon, Ultra 4; UFC801008) according to the manufacturer’s instructions. Concentrated filtrate was then supplemented with 4X Laemmli loading buffer boiled for 5 min and loaded onto a Tris-glycine 4–20% gradient gel (Life Technologies, EC6028BOX). The gel was run by electrophoresis to one third of its total length and then stained with Coomassie brilliant blue.

### Coomassie gel for mass spectrometry

Gel was fixed for 1 h at RT in 45% methanol and 10% acetic acid and rinsed twice with double-distilled H_2_O (ddH_2_O). Staining was performed with Coomassie blue solution (3 g/l Coomassie Brilliant blue G-250, Thermo 20279, in 45% methanol, 10% acetic acid) for up to 3 h at room temperature. Following two brief rinses in ddH_2_O, the gel was de-stained overnight in 45% methanol and 10% acetic acid. Finally, the gel was rinsed twice with ddH_2_O and stored in 1% acetic acid. After whole lane tryptic digest, mass spectrometry was performed on a Thermo Orbitrap Velos Pro at the Proteomics Core Facility of EMBL Heidelberg.

### Rif1 interactome enrichment analysis

Mass-spec raw data were analysed as described in ref. 49. In brief, samples were analysed in duplicates (Rif1^+/+^) or triplicates (Rif1^FH/+^) and processed using MaxQuant LFQ 1.5.3.8. A student *t*-test was performed on data pre-processed with Perseus 1.5.3.2, and *p*-values were plotted against *t*-test ratio. Proteins enriched above 1.5 fold were considered as significant interactors. Known human PP1 regulators^43^ and substrates^50, 51^ were extracted, combined and subsequently mapped to the corresponding mouse orthologous proteins with BioMart^52^. Two hundred and eight mouse PP1 regulators/substrates were thus compiled. A Fisher’s exact test (two-sided) was used to check the mouse Rif1 interactome enrichment for PP1 regulators/substrates, and the whole mouse proteome (downloaded on 03.30.2016 from Ensembl, http://www.ensembl.org/) was used as the background set.

### Protein expression and purification

The Rif1 protein fragment CRI (2093–2190) was purified as described previously^22^. Briefly, hexahistidine-tagged Rif1 CRI was purified on TALON (Clontech) beads, and the tag was cleaved by TEV protease. Mutations were introduced using the QuikChange kit (Stratagene). The gene coding for the α-isoform of PP1 (residues 1–330) fused with an N-terminal TEV cleavable hexahistidine tag was synthesized by GenScript and subcloned into pMAL-c2G under control of the Ptac promoter. PP1 was expressed in *E. coli* BL21 AI (RIL) cells grown in LB medium containing 0.5 mM MnCl_2_ until OD_595_ 0.6, followed by 1 mM IPTG induction for 20 h at 25 °C. The pellet was resuspended in buffer containing 50 mM Tris-HCl (pH 7.5), 300 mM NaCl, 1 mM MnCl_2_ in the presence of protease inhibitor cocktail (Roche Applied Science), and disrupted by a microfluidizer. The cleared extract was loaded on a TALON resin and purified according to the manufacturer’s recommendations. The eluted protein was incubated at 4 °C with TEV protease overnight, dialysed against buffer A (20 mM Tris-HCl (pH 7.5), 200 mM NaCl, 1 mM MnCl_2_, 2 mM DTT, 10% Glycerol), and loaded on a heparin Sepharose column (GE Healthcare). Peak fractions collected after elution by a NaCl gradient were dialysed against the buffer A and stored at −80 °C. Inhibitor 2 was purchased from New England Biolabs.

### Co-precipitation assay

The Rif1 fragments CRI (2093–2190), CRII (2226–2340), CRIII (2340–2418), CRI+II (2090–2316), CRII+III (2226–2418) and CRI+II+III (2093–2418) were subcloned into a pGEX-06-P-1 GST expression vector between the *Bam*HI and *Not*I sites. All the constructs were sequence verified. GST-tagged proteins were expressed in *E. coli* BL21 AI (RIL) cells grown in LB medium until OD_595_ 0.6, followed by 1 mM IPTG induction for 20 h at 20 °C. The GST-tagged HEAT repeat region (residues 1–966) was expressed in insect cells using the MultiBac system^53^. The cells were resuspended in PBS containing 5 mM DTT and protease inhibitor cocktail, and sonicated. The cleared extracts were incubated with Glutathione Sepharose 4 Fast Flow (GE Healthcare) beads for 2 h with rocking at 4 °C. The beads were washed two times with PBS. The extract containing hexahistidine-tagged PP1 was prepared as described above, pre-cleared on glutathione sepharose beads, and incubated with beads saturated with the Rif1 fragments overnight, rocking at 4 °C. The beads were then washed 4 times with buffer containing 50 mM phosphate (pH 7.4), 500 mM NaCl and 5 mM DTT. SDS loading buffer was then added to the beads, after which they were boiled at 95 °C for 5 min. The samples were loaded on a 15% SDS-PAGE gel, followed by a transfer on a nitrocellulose membrane. Detection was performed using a primary antibody against the hexahistidine tag with corresponding Alexa532 secondary antibody (Life Technologies). Membranes were imaged using a Typhoon Trio imager (GE Healthcare).

### Analytical gel fitration

Chromatography was performed on a Superdex 75 column in buffer containing 20 mM Tris-HCl (pH 7.5), 200 mM NaCl and 2 mM DTT, 1 mM MnCl_2_. The molecular weight values were calculated by interpolation from the standard curve obtained using a set of standard proteins.

### Isothermal titration calorimetry

Rif1 CRI and PP1 were dialyzed overnight into a buffer containing 20 mM Tris-HCl (pH 7.5), 200 mM NaCl, 1 mM MnCl_2_, 5 mM β-Mercaptoethanol, 10% Glycerol. CRI (140 μM) was titrated into PP1 (18 μM) using an iTC200 micro-calorimeter (Microcal) at 25 °C.

### Surface plasmon resonance

Measurements were conducted on a Biacore 3000 (GE Healthcare). PP1 was diluted to 30 ng/µL in 10 mM acetate (pH 5.5) buffer containing 1 mM DTT and 2 mM MnCl_2_, and covalently coupled to a CM5 sensor chip CM5 following the manufacturer’s protocol. Rif1 CRI and CRI mutants, were diluted with running buffer (10 mM Tris-HCl (pH 7.5), 250 mM NaCl, 1 mM DTT, 2 mM MnCl_2_, 0.005% Tween 20). Samples were injected over the PP1 and control instead of reference surfaces at a flow rate of 20 µl/min. Non-specific binding was controlled by subtracting the signal obtained on the control surface. Regeneration of the surface was achieved by a short injection of 2 M MgCl_2_. Each binding experiment was repeated twice. Kinetic parameters were determined by curve fitting with BIAevaluation software (GE Healthcare) using a simple 1:1 binding model.

### Nuclear magnetic resonance

The Rif1 CRI fragment was ^15^N or ^15^N/13C labelled by growing cells in M9 minimal medium containing [^15^N]ammonium chloride and [^13^C6]glucose as a sole nitrogen/carbon source. The protein was concentrated to 100–330 µM in an NMR buffer (50 mM sodium phosphate, 200 mM NaCl, 1 mM DTT, pH 6.8). To analyse the interactions, the complex of PP1 and ^15^N or ^15^N/^13^C labelled CRI was purified on a Superdex 75 gel-filtration column (GE Healthcare) in the NMR buffer and concentrated. NMR measurements were performed at 10 °C on Varian spectrometers operating at a ^1^H frequency of 600 or 800 MHz. The ^1^H-^15^N heteronuclear single quantum coherence (HSQC) spectrum of CRI was assigned using a set of triple resonance experiments: HNCO, intra-residue HN(CA)CO, HN(CO)CA, intra-residue HNCA, HN(COCA)CB and intra-residue HNCACB^54^.

### Dephosphorylation reactions

40 nM PP1 was premixed with interacting protein(s) in assay buffer (50 mM Tris-HCl (pH 7.5), 200 mM NaCl, 1 mM MnCl_2_, 2 mM DTT, 0.1 mg/ml BSA) and incubated at room temperature for 1 h. Dephosphorylation reactions were initiated by addition of 1 mM pNPP. Optical absorbance of dephosphorylated pNP product (405 nm) was measured at 90 s intervals for 90 min to determine initial reaction rates (V_o_).

## Electronic supplementary material

Supplemental files

Supplemental table 1
