# Supplementary material for: Mouse Rif1 is a regulatory subunit of protein phosphatase 1 (PP1)
Source: Sci Rep. 2017 May 18;7:2119. doi: 10.1038/s41598-017-01910-1 (PMC5437018; doi:10.1038/s41598-017-01910-1)

SUPPLEMENTAL FILES

Mouse Rif1 is a regulatory subunit of protein phosphatase 1 (PP1)

Rasa Sukackaite, Daniela Cornacchia, Malene Ringkjøbing Jensen, Philippe J. Mas, Martin Blackledge, Elin Enervald, Guangyou Duan, Tania Auchynnikava, Maja Köhn, Darren J. Hart and Sara B.C. Buonomo

SUPPLEMENTAL FIGURE 1. The majority of Rif1 fails to be solubilised by standard protocols. (**A**) Schematic representation of the position of the putative PP1 docking motifs in mouse Rif1 protein. (**B**) Only a minor fraction of total Rif1 can be solubilised by medium salt (0.4 M NaCl) extraction from ESCs nuclei[46]. (**C, D**) Rif1 from ESCs is completely refractory to solubilisation through nuclease-mediated chromatin digest. (**C**) Ethidium bromide-stained agarose gel showing different degrees of chromatin fragmentation obtained by treatment with different nucleases: micrococcal nuclease (MNase) for the indicated times; DNAse I (D); RNAse A (R); Benzonase (B) for 2 h. (**D**) Protein fraction obtained by the chromatin treated with a combination of DNAse I and RNAse A. Although no visible DNA is left, Rif1 is not released in the soluble fraction. (**E**) About 50% of Rif1 is released in the soluble fraction upon 3 cycles of snap-freezing in liquid nitrogen and thawing of nuclei isolated from both ESCs and mouse embryonic fibroblasts (MEFs). (**F**) Coomassie blue-stained SDS-PAGE showing the amount of GST-Rif1 fragments bound onto glutathione Sepharose beads. (**G**) Western blot of the samples shown in E, probed with anti-GST antibody. (**H**) Normalised NMR intensity ratio of the spectrum of CRI with PP1 (I) and without (I^0) corresponding to the two NMR spectra shown in Fig. 2D.

SUPPLEMENTAL TABLE 1. (A) List of all proteins interacting with Rif1, showing the single as well as the averaged LFQ intensities. (**B**) Subset of lower-confidence interacting protein: although peptides are present in at least two out of the three $Rif1^{FH/+}$ ESC lines, no corresponding peptide is present in the negative controls. In order to calculate the ratio of LFQs, the 0 values have been replaced with 1, driving therefore the ratios to artificially high values. (**C**) List of high-confidence nuclear Rif1 partners, with an enrichment cut-off ≥ 1.5 above the negative control.

Supplemental Figure 1

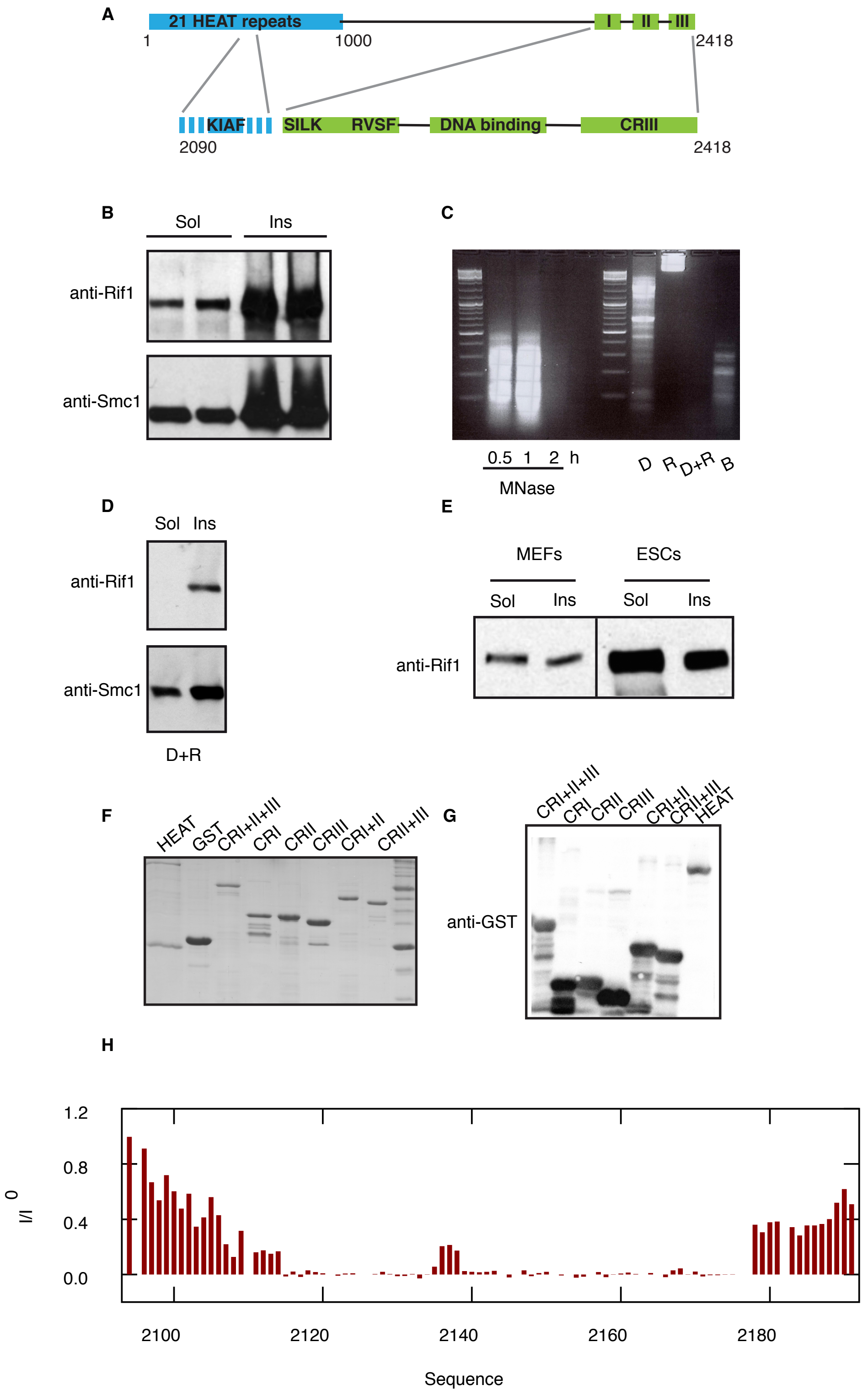

Supplement: Supplementary file 1 — Supplemental files [file 41598_2017_1910_MOESM1_ESM.pdf]
